# Dealing with complicity in fieldwork: Reflections on studying genetic research in Pakistan

**DOI:** 10.1111/1467-9566.13464

**Published:** 2022-03-24

**Authors:** Zainab Afshan Sheikh

**Affiliations:** ^1^ 4321 Centre for Advanced Studies in Biomedical Innovation Law Faculty of Law University of Copenhagen Copenhagen Denmark; ^2^ 4321 Centre for Medical Science and Technology Studies Section for Health Services Research Department of Public Health University of Copenhagen Copenhagen Denmark

**Keywords:** complicity, constructive complicity, ethnography, fieldwork, knowledge politics, methodology, Pakistan, power inequality

## Abstract

Health‐related ethnography undertaken in a context marked by social inequalities and colonial legacies requires critical attention to power imbalances in the fieldwork. In this paper, I draw on my own experiences from studying genetic research in Pakistan. As a Danish‐born female researcher with roots in Pakistan, I have followed genetic researchers and families dealing with genetic conditions in Pakistan. Through examples I unearth how encounters in the field were shaped by complicities of being in‐between the Danish and the Pakistani, of studying and doing international research at the same time, and of my inaction towards suffering families. I base my analysis on the notion that complicity manifests in a generative, and unavoidable, engagement with both complex structures of inequality and interlocutors. We can never fully understand the specificities or consequences of complicity—whether moral or epistemic—when entering, engaging with or representing our fields. However, by staying constructively with the tensions, instead of attempting to move beyond the discomfort that they might create, we can learn how to deal with the consequences and in that, build further the value of ethnographic activity.

## INTRODUCTION

*Am I creating precarious hope [by going into the homes of families]? Am I creating more of the same problems that I am studying and want to shed light on? Am I being instrumental in causing [yet more] suffering through global research, enhancing exploitation and harm?* (Field note, Multan, February 2017)

I posed myself these questions after an emotional day of fieldwork in the rural areas of Multan, Pakistan. As a Danish‐born female researcher with roots in Pakistan, trained in Public Health Science, I was following genetic researchers while they collected blood samples, biopsies and other health‐related information from Pakistani families. These families were affected by different types of genetic conditions, some of which were incapacitating and fatal. My was to understand how both families and researchers engage in genetic research, when this research is led by laboratories located in the global North. After weeks of fieldwork among people living lives characterised by poverty, serious health conditions and lack of healthcare, I realised that my engagement with the people I studied needed critical attention. Together with genetic researchers, I was welcomed into family households, people that were hopeful about what we could do for them. Knowing that we would probably never meet them again, after having collected data for our respective research projects, forced me to think about my role as a researcher. Ethnographic researchers are always confronted with the question of how our interaction with research subjects shape the field of study. However, when studying disease‐related issues in populations living with limited resources and limited access to health care the researcher can uphold or contribute to various harms related to sustained power inequalities (see Atkinson & Heritage, 1984; Clifford, 1983; Coffey, 1996; Pereira, 2017).

In this paper, I surface some of the methodological politics of contemporary ethnographic research on biomedical topics in resource‐poor contexts, by engaging the concept of *complicity*. Rather than looking at complicity in fieldwork as an act of violence rooted in moral deviance, I base my analysis on the notion that complicity manifests in a generative, and unavoidable, engagement with both complex structures of inequality and interlocutors. Inspired by Spivak’s (1999) concept *constructive complicity* I reflect on fieldwork encounters and how they shaped knowledge production in the context of my research. I argue that we can never fully understand the specificities or consequences of complicity—whether moral or epistemic—when entering, engaging with or representing our fields. However, as I will demonstrate in what follows, it is my contention that by facing and thinking through the complicities, instead of attempting to move beyond the discomfort that they might create, we can learn how to deal with the consequences and in that, build further the value of ethnographic activity.

In what follows, I will describe my methods and the setting for exploring data collection practices in Pakistan. Then I carve out my conceptual perspective on complicity before I present what it might look like in action, through reflecting on my position as a fieldworker: I dwell on the methodological implications of being a Danish‐Pakistani, doing research among people that I consider ‘my own’ but from whom I differ in significant ways. I also reflect on the way I visited family households to collect data together with genetic researchers. Lastly, I explore the challenges I experienced when realising that I could not do much for suffering families that were hopeful about the ways I could help. The paper serves to emphasise how focusing on the political dynamics of power and inequality in the researcher position are crucial for understanding the circumstances for knowledge production in the sociology of health and illness.

## THE ETHNOGRAPHIC FIELDWORK: METHODS AND SETTING

The genetic research that I studied involves multiple international collaborations between a laboratory located in Faisalabad, Pakistan and several high‐income laboratories located in Europe and the United States of America. My connection to the research laboratory in Faisalabad was established through a laboratory located in Copenhagen, Denmark. The Pakistani laboratory had attracted international attention in the field of genetics, due to the context of its being home to large accumulations of genetic conditions that in most countries are considered rare, such as microcephaly and ataxia. The large accumulations in Pakistan are rooted in cultural forms of kinship, where people get married within their families and casts, which leads to higher rates of genetic conditions (Shawky, 2013). The genetic research undertaken in these international collaborations is based on the collection of genetic material and health‐related information, in over 70 different disease categories, that is then analysed to understand the functioning of specific genetic variants and the genome in general.

My perspective on data collection practices in Pakistan builds on a tradition of studying research that is Western‐led while ‘locally’ implemented in resource‐poor settings (Anderson, 2002; Biruk, 2018; Geissler et al., 2008). This tradition has included perspectives from bioethics, a perspective that challenges the use of bioethical principles in local settings, postcolonial readings of data collection practices and perspectives on the social life of data collection in practice. I decided to undertake this research, as a PhD project, to focus on individual longings, social lives and modes of organisation involved in data collection (Sheikh, 2020).

Pakistan is a so‐called lower middle‐income country with a population of over 200 million people. Significant levels of poverty‐related vulnerability and illiteracy meant that data collection took place in a context characterised by limited access to medical facilities, and generally low and uncertain incomes (Kumar & Bano, 2017). I travelled around six different cities in Pakistan, both urban and rural areas, during 5 months of fieldwork in two periods; from December 2015–January 2016 and from February 2017–April 2017. Genetic researchers tracked down families with a wide range of autosomal recessive conditions by asking attendees at local mosques, servants or others who knew the local people well. A crucial part of my ethnographic fieldwork was to be present during collection trips to observe and engage in typical everyday conversations with geneticists and families, in order to understand the tacit knowledge of data collection practices (Madden, 2010). I also conducted interviews with researchers and with families enrolled in research. All in all, I observed 45 encounters between geneticists and families. I conducted 36 interviews with families and 15 geneticists, two medical doctors and two research laboratory leaders.

I have pseudonymized informants so that a person's statement cannot be traced back to them. I obtained ethical clearance from relevant institutions to conduct the research^1^ and verbal consent from families to conduct interviews and take and publish photographs. While making ethics protocols was an anticipatory exercise taking place in advance of the research itself (see Douglas‐Jones, 2021), as I will show with empirical examples, the practical dimension of research ethics was fluid and the result of multiple negotiations in the field. But first I turn to the conceptual framework, where I bring together perspectives from the fields of sociology, anthropology, philosophy, postcolonial studies and science and technology studies (STS) to carve out how a focus on complicity can help unpack fieldwork experiences.

## COMPLICITY IN THEORY: ENGAGING KNOWLEDGE POLITICS

Complicity is described as both a violation (which is the most common use of the word) and a companionship (see Marcus, 1997: 85). Even though these seem like clear opposites, both descriptions are relevant for understanding how I use the concept to discuss the politics of fieldwork encounters.

My engagement with the empirical field was itself embedded in the inequalities and power relations I sought to study. In this lies a complicity. In her study of the politics of unequal encounters in a global world, Sara de Jong (2009) has, drawing on interviews with women NGO workers, demonstrated that there cannot be a stable foundation for understanding unequal encounters, apart from the repeated acknowledgement of complicity. Based on scholar in feminist philosophy Charlotte Knowles’ (2021) phenomenological exploration of the notion of complicity, structural injustice and responsibility, this can be classified as structural complicity: a complicity with complex patterns of social interaction that systematically privilege some while disadvantaging others (p. 226). This classification entails that researchers from the global North, engaging with research participants from the global South, can be violating without intent. During fieldwork, however, the researcher's power is not only given, it is also negotiated *in situ* and thus has an open‐ended outcome (Scott, 2012). This means that the researcher can, by actively engaging one's own complicity, work towards resisting the harms of being at the privileged end of asymmetrical positioning in the world, through a type of companionship with interlocutors (see also Hellman, 2019; Marcus, 1997). Thus, the researcher is in a generative engagement with complex structures and interlocutors that itself changes the conditions under which it operates and has consequences for both what we consider to be right/wrong (the moral) and how we can learn about the field of study (the epistemic).

In *A Critique of Postcolonial Reason* literary and postcolonial theorist Gayatri Spivak (1999) defines *constructive complicity* as the only theoretical option to deal with the heritage of the past from a postcolonial perspective (see also Davis & Walsh, 2020; Kowal et al., 2011). Taking Kant's work as a departure point, she urges a postcolonial reading that denies neither the importance of, nor the problematic claims of, Western philosophy. She claims that researchers should find a place between accusations and excuses, staying constructively with the tensions, to avoid a ‘disabling’ complicity characterised by passivity. I transfer this insight to my own ethnographic position: as a researcher doing field studies in a postcolonial context, I argue for a constructive complicity that, within global research among resource‐poor populations, avoids a simplistic binary position between cruel exploitation and scientific victory. With this approach the point is not to seem forgiving of the violations to which researchers might contribute. The point is rather to know about potential harms, through a focus on responsiveness and transparency, ultimately to imagine the researcher as a situated knower involved in collective world‐making (Haraway, 1988).

Bringing the field of STS and postcolonial studies together, Sandra Harding (1998) has called for over‐advantaged groups to critically reflect on their privileged position and their relation to the oppression of other groups. This also involves grappling with emotional reactions: In line with what Renato Rosaldo (1993), an influential anthropologist, has highlighted, the process of knowing involves the whole self. He stresses the role of emotion, and states that there is no such thing as a detached, neutral or impartial position: to know we must also feel. Thus, emotions both indicate and help me unpack my complicity with interlocutors and complex structures of inequality. Importantly, however, the influence of the researcher can never be *completely* known or understood (Rose, 1997). This contingency of method has been thoroughly described by John Law (2004) in his book *After Method: Mess in Social Science Research*. He claims that methodology is not, and could never be, purely technical: ‘Events and processes are not simply complex in the sense that they are technically difficult to grasp (though this is certainly often the case). Rather, they are also complex because they *necessarily exceed our capacity to know them*’ (p. 6). I propose to understand the researcher's role in sustaining global inequalities in the light of this. Law (2004) argues that, by actively unmaking our desire for certainty and stable conclusions, we can gain insights to new ways of thinking about methods and the intent behind them.

How then, do we get to know about the complicities that shape knowledge production? I propose that it can be understood by ‘sitting with the mess’ (Lenette, 2020) or as Donna Haraway (2016) has put it ‘staying with the trouble’. When analyses based on fieldwork reach the readership, these analyses can seem polished and free from complicities. However, it is a messy and entangled approach that enabled my research process. In the following, I will reflect on how my interaction with the field created moral dilemmas and emotional reactions along with analytical and practical potential.

## COMPLICITY IN ACTION: UNPACKING FIELDWORK EXPERIENCES

In each of the following three sections I unpack experiences from the field followed by a reflection on how my fieldwork was constructively shaped by complicity. In the first section I reflect on the implications of entering the field through international connections as a Danish researcher with Pakistani roots; what I discuss as the complicities of the *in*‐*between* position. In the second section, I reflect on the implications of visiting family households together with geneticists in order to collect data. I discuss the complicities of being in the tension between doing global research while critically studying the same phenomenon. In the third section I reflect on the implications of leaving hopeful families dealing with deep poverty and disease. I discuss these as complicities of inaction.

### ‘From Pakistan but not *from* Pakistan’: Complicities of the in‐between

As described earlier, I arrived in Pakistan to understand how geneticists and families engage in international research through my own international connection: an already established genetic research collaboration. For each time I visited the laboratory in Faisalabad, the laboratory received monetary compensation (financed through my own funding budgets) for hosting me, and for facilitating my logistics and my interviews with families and with its own researchers. Rendering this more complicated, my background—born and raised in Denmark as a child of immigrants from Pakistan—shaped my field encounters in significant ways. I was described as being ‘from Pakistan but not *from* Pakistan’ by one of my interlocutors. This ambiguous position has similarities to so‐called ‘expatriate’ research (Kimura, 2016; Soh, 2008) or ‘halfie’ research (Abu‐Lughod, 1991; Subedi, 2006). Both of these concepts juggle the complexity of studying a community where one is a native or has a deep connection to the geographical and cultural place but simultaneously has a connection to another place (see also Koobak & Thapar‐Björkert, 2014).

As a scholar doing ethnography in Pakistan, I was ‘betwixt and between’ the Pakistani and the Danish (see Turner, 1969). Having travelled back and forth between Pakistan and Denmark for my entire life, I had a ready‐made relation to the country before entering the field. For me, thoughts of ‘leaving home’ and ‘arriving in the field’ (see Lien, 2015) were rather ambivalent, as I feel at home both in my parents’ and in my parents‐in‐laws’ residence in Lahore. I used these properties periodically during research. In most of the fieldwork situations it was easy for me to engage with cultural practices. Danish is my first language, but I speak Urdu and understand Punjabi. This meant that I could chat informally with interlocutors during my fieldwork and I did not need an interpreter to understand most of them. I would wear traditional Pakistani clothes when in the field to accommodate cultural norms, as I would in my personal life for particular occasions or family holidays to Pakistan. With this, and because of the colour of my hair and skin, I blended in and could disappear in the streetscape. I could thus appreciate many nuances in participant observations, and foster closer relationships with local people: The more one is like the participants in terms of culture, gender, race and socio‐economic class the more it is assumed that access will be granted and meanings shared (Merriam et al., 2001).

Despite being accustomed to many aspects of Pakistani culture, I do not consider them ‘givens’ in my own life. When I was unsure of what to do, I mirrored what other female researchers did: In rural areas other female geneticists, who do not normally cover their hair, would loosely wrap a shawl around their head when entering the private household of a family. My attempts to ‘mirror’ this type of conduct brought with it some awkward moments, for example during one interview where a large shawl kept sliding off my hair. Again, I was used to these kinds of customs in my personal life, to show respect, especially in front of elder distant relatives; however, not in a research situation.

It was not always an option to blend in. Differences between myself and my interlocutors were also noticed and articulated both during interviews and during participant observation. For example, many interlocutors noticed that I have a different accent when speaking Urdu, and borrow words from English to fill in gaps in my vocabulary (the ‘borrowing’ of English words is a common practice based on colonial legacies of cultural influence (Sipra, 2013)). I have only been schooled in reading and writing Urdu up to sixth grade, thus my writing is equivalent to middle‐school‐level in Pakistan. It was a surprise for the researchers at the laboratory to know that I am not fluent in written Urdu. A friend and laboratory member, Zaid, remarked that it was odd that I, ‘being a Pakistani’, did not have the basic writing abilities. Evidently there were certain expectations to being a Pakistani (scholar) that I did not fulfil. During dinner one evening, one of the researchers, Annam, was talking about her mother's health issues. To explain her situation, Annam said: ‘You come from a solid system. Our worries here are different’. In another case, when it came to beliefs, I was dubbed a stranger by Haider, a father of three children with genetic conditions: ‘In our community, as you probably don't know, people believe in the detrimental force behind *buri nazar* [evil eyes]’. In these situations, I would acknowledge my position as an outsider who wanted to learn more about their thoughts. Thus, at times I felt a strong sense of community, namely, that I was doing research ‘among my own’, while at other times I was distanced from the informants.

What do these moments from the field say about the complicities of my in‐between position? Across a range of empirical sites such as the biobanking of indigenous blood (Radin, 2017), medical research participation in sub‐Saharan Africa (Kingori, 2015) and the Human Genome Diversity Project (Reardon et al., 2015) ethnographic research has highlighted the ways in which global inequalities shape data collection for the life sciences. Inequalities were also shaping my own data collection. My privileged position within global hegemonic structures conditioned my advantageous research opportunity. I arrived in the field to study how people relate to resourceful distant actors whilst being one of those same actors: coming to Pakistan as part of research born in the global North. As a ‘situated knower’, I had a particular position in the field (see Harding, 1991) marked by a complicity with complex structures of global inequality in health, wealth and research that granted me access to the field of study.

My position as being in‐between the Danish and the Pakistani, an ‘expatriate’ researcher, generated particular circumstances for knowledge production. In many ways I was in a constructive engagement with complicity through my relation with interlocutors. Having a ready‐made relation to Pakistan, knowing the language and the cultural cues led to unique insights about the people I studied. I could ask sensitive questions as I, being a specific type of stranger (see Simmel, 1950), was ‘excused’ for bringing up matters, such as cultural practices of kinship, gender‐roles and patriarchal structures. These matters could perhaps be taken for granted by both interlocutors and researcher, if it was conducted by a more local ethnographer. I could engage with my interlocutors in a respectful manner. My ambiguous position of familiarity/strangeness could both emphasise and conceal certain sides of me. My Pakistani heritage could mask my Danish upbringing. Sometimes I used this strategically to downplay my own socio‐economic background from that of those I was studying, disguising my privilege through choice of clothes, in order to ‘blend in’ in interview situations. While ‘blending in’ as a means of showing respect and fostering mutuality can give unique access to life worlds it also actively conceals power relations between the researcher and the researched. Blending in, in the same manner I did in my personal life, proved constructive for my research; however, it is important to consider how it might reinforce structural and interpersonal inequalities to disguise power: after all, it is a move of power in itself.

Importantly, however, as in the situation with my writing and speaking abilities, I could not control how I was understood by interlocutors. Thus, my in‐between position was ambiguous, constructive for important aspects of research. At the same time, what might be constructive for research insights should also be considered in terms of broader structural conditions.

### ‘So, we are here to take his sample’: Complicities of doing what you study

During fieldwork one afternoon, I arrived at an apartment in one of the many middle‐class areas of Faisalabad with three geneticists: Ali, Numan and Alia.

*As we walked in, I was guided to a small living room. In this living room, clearly reserved for guests, old British furniture stood next to each other and decorated chairs and a couch were placed at the centre of the room. A male household member brought tea and biscuits and placed them in front of me. Numan, a geneticist, started explaining to me; ‘So we are here to take his sample to see if we can trace any genetic condition. They’re dealing with infertility’. I nodded and greeted the well‐groomed man who was in his 40s and smiling politely to me. I explained that I came as a Danish scholar to observe and ask about their experiences of research participation, with eager nods in return. The man, Assad, reassured me that I was more than welcome: ‘I am happy that you [directed to me] are here. My wife and I have tried everything to have our own children, now you are here to see us. Maybe this will help’. As Ali and Numan asked about fertility problems in the family, Alia started drawing a family pedigree*. (Field note, Faisalabad, January 2017)

This encounter demonstrates a number of tensions around how a family could read my role. My presence was never unnoticed. I was often introduced by the geneticists as ‘the *baji* [sister] from Denmark, doing research on people's hopes and concerns.’ To obtain consent from the families, I would repeat my purpose of joining the researchers. Not one family during my entire fieldwork denied me entry or permission to observe and potentially ask questions. Despite some families having general worries about the misuse of data I was treated as a guest of honour. After having observed the geneticists’ data collection, I would go to another room to interview family members when this was a possibility. This means that Pakistani families were effectively enrolled in two projects: genetic research and my own ethnographic study. In the above case, I met Aliya in the next room. She looked at me, smiled and said: ‘I’ve heard that you are here to ask me some questions’. At first, I was a ‘guest’ observing the data collection of genetic researchers and then I was moved to a different location, to do my own (even though observation was also part of my data collection): an interview about their experiences and concerns about genetic research and their infertility. A geneticist would often join my interviews. I could not control what the geneticists learned about the families, in the same way that the geneticists could not control what I observed during their data collection. This could potentially undermine families’ chance to differentiate between my role and that of the geneticists while at the same time strengthening my relation with geneticists and our understanding about each other's research.

In many instances, however, my engagement with families also opened a space of mutual dialogue and a platform for families to share their thoughts and concerns (see Sheikh & Hoeyer, 2019; Sheikh & Jensen, 2019). Geneticists realised there were questions that families wanted answers to. Through my interview I could facilitate further conversations between the two parties. It could for example be in cases where the family did not fully understand the link between the genetic condition and intra‐family marriages. On occasions, it became unclear who was joining whom: were the genetic researchers joining me in my quest to gain access to families’ accounts of vulnerable and intimate aspects of their lives, or was I joining the geneticists?

Another way that my field encounters created a platform to potentially share thoughts and concerns was when taking photographs. I took photographs during my last trip to Pakistan to portray fieldwork, while the geneticists took photographs with the purpose of documenting clear phenotypical traits (for example smaller‐than‐average head circumference or a skin condition). I found that adherence to ethical protocols and practices were rarely sufficient when it came to attend to the many types of social environments in which ethical issues unfold. This means that I took photographs based on a negotiation between the families and myself. I would for example ask: ‘What can I take a picture of?’ to respect people, including cultural boundaries of *parda* [segregation by sex and veil (cf. Shaw, 2009)] instead of taking pictures of what I found interesting. Photography is just as much a subjective representation of reality as text (Soukup, 2014); however, the family could see and relate to photographs in a different way. They could also approve of them immediately on my smart phone. Given my entry to the field with geneticists, people could still have felt unable to deny my desire to take photographs. Therefore, I chose to blur the faces of children and family members with mental impairments, who could not understand why I was there. Some asked me to take pictures ‘to show others’ how they lived, while some would gather the entire family for a portrait. Raheela, mother to a blind and disabled girl, pointed to her daughter and said: ‘Take a picture while she's drawing, she really enjoys drawing. I want you to show people that we are doing okay.’ One man, Faisal, asked me to photograph his cousins (who had microcephaly) exactly as the genetic researchers did. These examples show how differently people could perceive my role and presence. I have shared these photographs with audiences in presentations and in my PhD dissertation, but given the conditions for data collection I have experienced difficulty in featuring the photographs in presentations and papers in a manner befitting the wishes of participants.

How do these fieldwork experiences surface my complicities of doing what I studied? In the sociology of health and illness, researchers often depend on, or even validate, medical practices they are studying, and might be critical of. With ‘doing what I studied’ I refer to the fact that the mode of collecting data for my research followed the same pattern as the geneticist's data collection. I depended on, and conducted, the same practices that I was studying with a critical perspective. My engagement with the field and knowledge production was in many ways conditioned by the way I entered and left family households with geneticists. Sociologists doing fieldwork have discussed the tension that arises between being part of both a disciplinary field that calls for a critical perspective on the people who one has studied, and an ethnographic field that values a sense of moral obligation towards research subjects (Anspach & Mizrachi, 2006: 714). As I was studying the workings of an international research project, a critical perspective on the genetic research practices necessarily meant a critical perspective on my own practices in my own international research project. As the reflections from the section above show, my data collection was intertwined with that of genetic researchers. The genetic research laboratory in Faisalabad enabled my research to take place. At times, I seemed to also enable theirs. Similarly, my moral obligation towards research subjects also became a question of the integrity reflected in my own research practices. I tried to be aware about this parallel element in my work. How could I raise critical questions about data collection practices in Pakistan, when I was feeding off the same? Were my own research practices really validating medical practices that I may be critical of? Instead of hiding my complicity, I asked myself questions like this during fieldwork.

My engagement with researchers was constructive, in that we were focused on engaging with families for our respective research goals (see Bosk, 2003). Geneticists were collecting blood samples, asking about family histories and taking photos and I was doing similar things for my own research interest. We were collecting data to make our way in the world, mediated through various institutions and academic disciplines. Through joining my interviews geneticists were also able to understand my project better. At the same time when I interviewed and took photographs of families I could mediate what they wanted to communicate to the geneticists and the world; what can be tagged a constructive complicity with families. I actively sought to generate a representation of the people in the field that *they* wanted. Still, there was a clear tension between my using interlocutors for research and being useful myself for the genetic researchers and families. My easy access to family households, to interviews and to taking photographs reflects the hierarchies and particular position of authority that I was benefitting from. I had to think through the consequences of my position, even when, for example, emphasising pictures as a part of a family's agency in channelling important messages to others that would otherwise be silenced. Over time, there has been a persistent critique of taking and using pictures of people experiencing suffering for communicative purposes (Wilkinson & Kleinman, 2016). There *is* undisputedly a fine line between wanting to use pictures for research purposes and to exploit people who are already living precarious lives.

Thus, my position as a researcher who was doing what I studied, give important insights into the politics of knowledge production: Instead of assuming an act of violence in complicity committed by ‘others’ (which speaks to a global exploitation narrative), we as social science scholars engaged in fieldwork, need to sit with the ‘mess’ of being complicit ourselves. Critical questions to the field and critical questions to one's own fieldwork can go hand in hand and foster a constructive complicity.

### ‘I just feel like throwing up’: Complicities of inaction

At times, I could feel overwhelmed by engaging families marked by disease, poverty and lack of healthcare. Some did not have enough food to eat or clothes to wear, and some were dealing with genetic disorders that were completely incapacitating. Some children were even close to death.

As can be seen in the field note where I enter the household of Assad, I was sometimes met with hope that I could help them with their situation. In one case, described elsewhere, a father even asked me to take his children with me to Denmark for medical help (Sheikh & Hoeyer, 2018: 175). This request marks the desperation of their situation and also an important aspect of my position: In cases of suffering, where participants lacked healthcare and basic necessities, my presence could even intensify people's hopes and expectations for a cure. In situations like this, I felt uncomfortable ‘dropping in’ to their lives to do my own data collection while not able to help people in the ways that were most urgent for them.

The discomfort often manifested in nausea when leaving family households. After having visited a family with two girls, blind and disabled, living in deep poverty, one of the genetic researchers, Zaid, offered to buy me lunch: ‘We can go to KFC, it's just 10 min from here, do you feel like doing that?’ he asked me. I had pictures in my head of the small shed they called home, could still smell the inadequate toilet facilities, and was replaying their stories about the girls having tried to set themselves on fire with matches because they did not want to burden their parents anymore. Visualising the burn injuries that one of the girls had shown me and realising my incapacity to do anything for them, I answered: ‘No, I just feel like throwing up.’ This answer surprised Zaid, but knowing me, he quickly understood that my reaction manifested the discomfort of having witnessed the situation of the family. I had strong reactions to witnessing health‐ and poverty‐related suffering at several other points during research.

The Pakistani custom of offering *chai* [tea] provides another example of this. When we visited households, as in the case of Assad, many of them had prepared tea and put biscuits on the table. ‘*Chai leh ao!* [Bring some tea!]’ is a common call to family members or servants in households when guests arrive. As it is considered impolite not to drink the tea offered, I would often drink several cups a day. During one visit, at the home of a family with several cases of schizophrenia, I was interviewing a man about his mother and sister, who were both psychiatric patients. During the interview, he began speaking about sorcery and evil‐spirited *jinns* (see Khan, 2006; Rytter, 2010). His eyes were wide open while he explained that his father had committed suicide and that it could be the only option sometimes. It dawned on me that he was also struggling with mental health problems. I felt overwhelmed and the tea started to make me nauseous. Despite being the tea‐lover that I am, this incident subsequently made me feel nauseous even thinking about tea. I also experienced nightmares and trouble sleeping during fieldwork. Despite having travelled to Pakistan many times, I had never witnessed the level of poverty or so many severe and untreated health conditions this close.

Genetic researchers collecting samples would help where they could during field trips. They offered wheelchairs and medicine when possible, and in many cases provided monetary compensation to families. This helped me navigate my own need to ‘do something’ when confronted with families’ living conditions and lack of health care. Leaving families in the conditions I witnessed without helping seemed wrong, a moral discomfort that was crucial for my choice to help them in the ways I did. When meeting families that were living in poverty, I chose to give them money at the end of the interview (approximately 2000 Pakistani rupees, which is equivalent to approximately 12 British Pounds). For some families this could amount to a quarter of a month's salary. Before entering the field, the thought of giving money after interviews was very far away from me, as I linked it to ideas about coercion. Fieldwork nuanced my perspective, although my choice was undeniably a ‘move of superiority’ as De Jong (2017) puts it. Many families did not have access to health care and were willing to take many risks due to their longing for a cure.

How does the emotional aftermath of witnessing suffering surface complicities of inaction? When a privileged researcher like myself enters the homes of people living in deep poverty and creates an unintended and precarious form of hope, it can reinforce the harm that lies in existing inaction towards resource‐poor populations in the global South. My data collection was for my own researcher purposes, and was not going to help people in the ways that were most urgent for them. In this lies a complicity. The discrepancy between my own interests and the families’ hopes about my interests, based on their life conditions, affected me emotionally during fieldwork. This shows in my bodily reaction when offered a meal after visiting a family. It shows with the nightmares I experienced. My reactions give insight into the emotional and embodied forms of knowing about my own position in the hierarchies I was observing during fieldwork (see Law, 2004; Rosaldo, 1993; Stodulka, 2015). For Sara Ahmed (2004), emotions are an important factor in understanding how structural violence operates under social and political conditions (see also Varma, 2020). I reacted strongly to what I experienced in the field when realising my inaction towards people's suffering, exacerbated by the fact that I was not used to witnessing poverty and suffering. Thus, nausea and nightmares are illustrative of my privileged life and power in the field. At the same time, the reactions also reflects powerlessness: Despite being in a privileged position, I too, was powerless in the face of suffering.

There was nothing ostensibly constructive in these particular moments of fieldwork. Did I here experience the limits of a constructive way of dealing with complicity during fieldwork? In one sense, yes. In instances of deep poverty and precarious hope, I could no longer navigate my way out of the moral ambiguity of being a privileged party in the field. One cannot always insist on a constructive complicity when in the field. Giving money to suffering families felt like the right thing in given situations, but it did not relieve my moral discomfort. Traditionally the concept of ‘benefit sharing’ addresses questions about how gains and resources in an international project should be shared among researchers, participants or host institutions, who have contributed to the research to avoid increasing the gap between high‐income and low‐income countries. Studies have shown that what study participants and researchers consider a fair mode of sharing benefit is in no way straight forward (Kingori, 2013; Molyneux et al., 2012). In my limited attempt to deal with power inequalities, I was feeding into it at the same time—a fragile engagement with families that could arguably be reinforcing neo‐colonial practices of ‘aid’.

At first, my own reaction to potentially exploiting people that were already suffering was characterised by passivity due to the emotional implications of being in the field. To understand the families’ situation it was important that I was present and close enough to sense their desperation. However, it was difficult reaching a distanced sense of the field, which is important for starting the analytical process (see also Scheper‐Hughes, 1993). Finding a place between closeness and distance, to foster analysis and to avoid a ‘disabling’ complicity, proved difficult in practice (Spivak, 1999). In order to overcome passivity and do the analysis, time was crucial. With time, my emotional reactions became constructive for my academic work. It helped me generate a strong focus on the interlocutors’ hopes and concerns. It gave me a different sense of responsibility towards the people that I studied. In fact, my work became heavily focused on the families’ perspectives on the research encounters. Several scholars have argued that it is important to restore a sense of moral purpose and solidarity in social science that might stimulate both critical thought and social action in relation to social suffering (Biehl, 2016; Briggs & Mantini‐Briggs, 2016; Wilkinson & Kleinman, 2016). Nancy Scheper‐Hughes (1993) has emphasised that social scientists are never neutral observers, but rather part of a politics of representation through their own descriptions.

My emotional reactions shaped the politics of my own knowledge production. It was a constructive engagement with the complicity of my inaction in the field that shifted my focus to the broader context of representation. Through my research I attempt to give voice to what my informants *find to be relevant*, to address what is most important in their lives (see Sheikh & Wahlberg, 2021). Thus, constructive complicity is not only limited to the fieldwork encounters, it can also mean the unsurfacing of inequalities that are otherwise elided, thus it extends to the politics of representation and has a temporal dimension.

## CONCLUDING REMARKS: IMPERFECT ATTEMPTS OF DEALING WITH COMPLICITY

Returning to the opening field note of this paper that captured some of the questions I was battling with during fieldwork; was I creating precarious hope, enhancing exploitation and harm through my research? As I have shown in this paper, it is not possible to answer questions about one's own engagement in the field as a researcher with binary questions. The questions from the field note should rather be thought of as an example of doubt and moral unease that should be shared in the academic debates that theorise research engagement.

The contribution of this paper is a call to engage with knowledge politics in the sociology of health and illness. I have studied the politics of my own research, by unpacking my fieldwork among geneticists and families dealing with genetic conditions, undertaken in a context of global inequalities, colonial legacies and power imbalances. I have synthesised a range of experiences within three important aspects of my position: my in‐between position as a Danish researcher with Pakistani roots, my position of doing global research while critically studying the same phenomenon and my inaction towards peoples’ suffering.

Spivak’s (1999) concept of constructive complicity stresses the importance of the researcher staying constructive with tensions related to questions of sustained inequality, in order to avoid a complicity that disables research. This helped me to avoid reaching ‘black and white’ conclusions about my own position. My position was ambiguous, constructive for important aspects of research, sometimes also for the interlocutors, but not always in terms of broader structural inequalities in health, wealth and research. As I have shown, in my own research, I worked with different imperfect attempts to address power inequalities through my practice. Attempts to deal with complicity will always be imperfect and do not always resolve the moral discomfort or the problems at stake. It is not clear what hopes or harms are left behind. Complicities cannot be neatly resolved. What then, do we gain from this ‘sitting with the mess’? When undertaking research among people living precarious lives in resource‐poor contexts—people who cannot speak for themselves due to global and institutional structures (Spivak, 2004)—it is critical for the ethnographer to consider how hegemonic conditions have consequences for all aspects of the research engagement. We can uphold dominance and inequality through research, and we can also work reflectively towards the opposite. For the ethnographer to critique global structures of inequality, one needs to acknowledge one's own position within them. By staying constructively with the tensions related to my own position during fieldwork, I could focus critically on the data collection practices, even when I was engaging in the same. In this sense, constructive complicity in field work can enable critique rather than oppose to it (see Montgomery et al., 2017).

Even though we cannot neatly resolve complicities, or fully know their implications, the question of what type of engagement with interlocutors who are dealing with poverty, disease and limited access to health care, we as social scientists can justify, needs more attention. It is part of a crucial and much needed academic conversation about research conducted among populations in the global South. I argue for a need to dwell more on complicity in the researcher position, and the stakes of the decision to engage in, ethnographic fieldwork among people living in medical and financial precarity, by taking the time to think through the mess rather than trying to overcome it. This might foster greater responsibility in work with vulnerable populations and ultimately enable us to envision a different world.

## AUTHOR CONTRIBUTIONS

**Zainab Afshan Sheikh:** Conceptualization (lead); Data curation (lead); Formal analysis (lead); Investigation (lead); Methodology (lead); Project administration (lead); Writing – original draft (lead); Writing – review & editing (lead).

## Data Availability

Participants did not consent to the sharing of their data, and as such the data supporting this publication are not publicly available.

## ENDNOTE

^1^
I obtained ethical clearance from the institutional committee of the host institution of the Pakistan‐based laboratory. Additionally, the research was approved by the Danish Data Protection Agency and the European Research Council.
